# Diagnostic multiparametric models and antibiotic practices in febrile infants younger than 90 days

**DOI:** 10.1007/s00431-025-06528-4

**Published:** 2025-10-09

**Authors:** Laura Martino, Anna Camporesi, Ilaria Lazzareschi, Gaspare Cascio, Francesco Proli, Giulia Bersani, Cristina De Rose, Giuseppe Zampino, Piero Valentini, Danilo Buonsenso

**Affiliations:** 1 School of Pediatrics, Università Cattolica del Sacro Cuore, Roma, Italia; 2 Anesthesia and Intensive Care Unit, ‘Vittore Buzzi’ Children’s Hospital, Milan, Italy; 3 Department of Woman and Child Health, Fondazione Policlinico Universitario A. Gemelli IRCCS, Largo A. Gemelli 8, 00168 Rome, Italy; 4 Area Pediatrica, Dipartimento di Scienza Della Vita e Sanità Pubblica, Università Cattolica del Sacro Cuore, Roma, Italia; 5 Medicine and Surgery, Università Cattolica del Sacro Cuore, Roma, Italia; 6 Centro di Salute Globale, Università Cattolica del Sacro Cuore, Roma, Italia

**Keywords:** Febrile infants, Fever, Procalcitonin, C-reactive proteine, Urine analyses

## Abstract

Most studies in febrile infants evaluated individual laboratory data to predict diagnoses of febrile infants, not taking into consideration the combination of data. In this study we aimed to analyze the diagnostic accuracy – both individually and in combination – of C-reactive protein (CRP), procalcitonin (PCT), white blood cell count (WBC), and neutrophil count (ANC), along with clinical findings, in predicting whether a young febrile infant has a viral infection, urinary tract infection (UTI) or serious bacterial infection (SBI). This is a retrospective, observational, single center, cohort study during a period of 7 years (2018–2024). A total of 615 patients were included in the study, of which 352 male (57.3%) with a median age of 51 days (IQR). For the detection of SBI, AUC values were greater for PCT (OR 1.130; 95% CI: 1.062–1.203, *p* < 0.001; AUC 0.75) than for CRP (OR 1.017; 95% CI: 1.008–1.026, *p* < 0.001; AUC 0.64). The comparison of ROC curves for CRP and PCT suggested no statistically significant difference between the two markers in detecting viral infections, UTI without bacteremia, UTI with bacteremia and SBI. The AUC values of the markers were always higher if used in combination with urinalysis and clinical findings. In addition, antibiotic sparing procedures were implemented in a third of patients with viral infections and oral shift frequently implemented, without negative outcome.

*Conclusion*: Our findings suggest that single biomarkers are not useful in discriminating viral and bacterial infections, but a combination of CRP (or PCT), urinalysis, and clinical findings, may be more accurate.

**Table:** 

**What is Known:** • *Febrile infants under 90 days of age are at high risk for serious bacterial infections, often leading to invasive testing and antibiotic treatment. However, single biomarkers like CRP or PCT have limited diagnostic accuracy.*
**What is New:** • *Combining biomarkers with clinical findings and urinalysis improves diagnostic precision, potentially reducing unnecessary treatments and supporting safer, individualized management strategies in this population.*

**Supplementary Information:**

The online version contains supplementary material available at 10.1007/s00431-025-06528-4.

## Introduction

Febrile infants younger than 90 days of age account for a high percentage of annual emergency department (ED) visits. Patients in this age group have a higher risk of developing a serious bacterial infection (SBI) and an invasive bacterial infection (IBI). SBI and IBI must be promptly identified and treated since they may be life-threatening. The management of these patients remains challenging, as they present with subtle clinical features, making it often difficult to recognise a clear focus of infection and consequently distinguish between infants with an early SBI and those with a benign viral infection. [1] Consequently, the management of febrile infants usually involves a more cautious approach, including performing invasive diagnostic procedures such as blood, urine and cerebrospinal fluid (CSF) collection, as well as empirical broad-spectrum intravenous antibiotic therapy once cultures are obtained. [1] This approach aims to minimize the risk of infectious complications. On the other hand, it frequently leads to unnecessary hospitalisation and treatment, resulting in potential iatrogenic impact. Moreover, although the prevalence of SBI still remains greatest in the first months of life [2], it has been decreasing as of late thanks to better prevention of perinatal Group B Streptococcal (GBS) infection and the and the widespread use of conjugate vaccines against *Streptococcus pneumoniae*, *Neisseria meningitidis*, and *Haemophilus influenzae* type b (Hib) [3]. For this reason, it may be necessary to reconsider traditional recommendations [4]. In recent decades, this increasing awareness has fostered research on new and old biomarkers and in how to better discriminate which young infants with fever benefit from a more thorough approach. [5] There are some easily available biomarkers that have been studied for their utility in clinical prediction rules, often in combination or together with clinical findings, to risk stratify febrile infants. They include white blood cell count (WBC), in particular absolute neutrophil count (ANC), C-reactive protein (CRP) and procalcitonin (PCT) [1]. However, most of the available literature is derived from datasets from North America and Northern Europe, while there is little evidence if a more conservative, biomarker-led approach, is feasible for Italian children.

The primary aim of our study was to investigate the diagnostic accuracy of biomarkers (CRP, PCT, WBC, ANC) in a population of febrile infants younger than 90 days of age admitted to our ED. Our secondary aim was to evaluate our experience in the management of this population to assess when lumbar puncture is necessary and investigate whether it is possible to reduce the duration of intravenous antibiotic therapies, in favor of oral ones, without negatively impacting patient outcomes.

## Materials and methods

### Study setting and design

This is a retrospective, observational, single centre, cohort study conducted on infants younger than 90 days of age admitted for fever at the ED of Fondazione Policlinico Universitario A. Gemelli in Rome (Italy), comprising a period of 7 years – from January 2018 to December 2024.

### Study population

Infants were included in the study if they met the following criteria: 1. ≤ 90 days of age, 2. Presented with fever, defined as an axillary or rectal temperature of ≥ 38 °C either at triage (measured by a triage nurse) or at home. We excluded preterm infants born under 33 weeks of gestational age and those with underlying pulmonary, haematological or immunological diseases.

The infants were defined as well appearing if they showed a normal general appearance, normal work of breathing, and adequate peripheral circulation. The duration of fever was determined by considering the time elapsed from the moment the family reported recording a temperature above 38 °C to the time of the first paediatric evaluation. The duration of fever was defined as one day if it had started within the 24 h preceding the evaluation.

In our hospital, it is standard practice for neonates with fever to undergo blood and urine collection for determination of WBC, CRP, and PCT, along with urinalysis and blood and urine cultures. Infants aged 29 to 90 days are also managed similarly, although more recently this approach is being called into question and decision making is left to the attending clinician. CSF collection for analysis is performed in the presence of meningeal signs on admission and/or during hospitalisation, deterioration of clinical condition, or significantly elevated inflammatory markers.

### Definition and outcome measures

We obtained demographic data, clinical history, physical examination and laboratory results (including complete WBC, CRP, PCT, urine chemical test, blood cultures, urine cultures, respiratory viruses’ antigen or PCR testing from nasopharyngeal swab and CSF chemical analysis and cultures) from the electronic medical records. C-reactive protein was considered abnormal if higher than 5 mg/L, and procalcitonin was considered abnormal if higher than 0.5 ng/ml. A panel of three expert in pediatric infectious diseases have assessed the final database, blinded to the clinical discharge charts, and classified the etiological diagnosis according to the following data. Based on that, infants were classified as affected by:

1. Viral infection in patients with detected viral infection on nasopharyngeal swab and microbiological exclusion of bacterial infections on urine, blood, CFS or other clinically relevant specimens, according to a recently proposed classification [6] When no viruses were detected, but patients had absence of criteria for bacterial pneumonia, they were classified as viral infections, based on comprehensive clinical, laboratory, imaging and microbiological data, still based according to a recently proposed classification [6].
2. UTI: defined by the growth of a single microorganism with at least 50 000 colony-forming units (CFU)/mL from a catheterised urine specimen or at least 100 000 CFU/mL from urine collected via a voided specimen, with or without a concomitant positive blood culture.
3. SBI:
  a Sepsis or bacteraemia: defined by the isolation of a single bacterial pathogen in the blood, excluding growth of bacteria commonly considered to be likely contaminant
  b Osteomyelitis or Septic arthritis
  c Bacterial meningitis: defined by the detection of bacterial pathogens in CSF

4. Undefined: if infants did not meet any of the previous categories.

This observational study involving human participants followed the ethical standards of the institutional and national research committee and with the 1964 Helsinki Declaration and its later amendments or comparable ethical standards. This study is a sub-analysis of a larger European multicentre study comparing admissions before, during and after the beginning of SARS-CoV-2 pandemic (ID 3497, Prot 0049226/20, 02/10/2020)).

### Statistical methods

Descriptive data are shown as absolute numbers and percentages for categorical variables, and median and interquartile range (IQR) for continuous ones. Normality of data was assessed with Kolmogorov–Smirnov test. Patients have been classified in one possible final diagnosis between “viral infection”, “serious bacterial infection”, “urinary tract infection”. For these outcomes, logistic regression models have been used to study the role of CRP and PCT alone and in combination with other symptoms on presentation. Adequacy of the models was assessed with Hosmer–Lemeshow test. Possible final models were evaluated comparing their respective Akaike Information Criterion (AIC) and Bayesian Information Criterion (BIC) where indicated. Models including CRP and models including PCT were then compared using the De Long’s method.

## Results

### Study population

A total of 615 patients were included in the study, of which 352 male (57.3%) with a median age of 51 days (IQR). 98.7% of patients were in good clinical condition on admission and the most frequently reported symptoms were nasal congestion (50.4%) and feeding difficulties (30.9%). The median duration of fever at the time of evaluation was 1 day.

361 (58.7%) patients were diagnosed as having a viral infection, 87 (4.1%) with a urinary tract infection, 21 (3.4%) with a serious bacterial infection and 146 (23.7%) remained undefined. Poor outcome only occurred in 1 patient with a final confirmed diagnosis of *Streptococcus agalactiae* meningitis.

The demographic and clinical characteristics of the study population are presented in Table 1.

**Table 1:** Demographic and clinical characteristics of the study population

	Study population (*N* = 615)	Viral infection (*N* = 361)	Urinary tract infection (*N* = 87)	Serious bacterial infection (*N* = 21)	Undefined diagnosis (*N* = 146)
Male	352 (57.3%)	195 (54.2%)	61 (70.1%)	17 (81.0%)	79 (54.1%)
Age (days)	51.0 (31.0–70.0)	41.5 (22.0–61.0)	18.0 (10.0–36.0)	45.0 (26.0–67.0)	45.0 (26.0–67.0)
Comorbidity					
Yes	79 (12.9%)	31 (8.6%)	25 (28.7%)	4 (19.0%)	19 (13.0%)
No	536 (87.1%)	330 (91.4%)	62 (71.3%)	17 (81.0%)	127 (87.0%)
Good clinical conditions					
Yes	607 (98.7%)	357 (98–9%)	86 (98.9%)	19 (90.5%)	145 (99.3%)
No	8 (1.3%)	4 (1.1%)	1 (1.1%)	2 (9.5%)	1 (0.7%)
Symptoms at the moment of evaluation					
Difficulty feeding	190 (30.9%)	131 (36.3%)	28 (32.2%)	7 (33.3%)	24 (16.4%)
Vomit	46 (7.5%)	26 (7.2%)	3 (3.4%)	1 (4.8%)	16 (11.0%)
Rhinitis	310 (50.4%)	249 (69.0%)	22 (25.3%)	2 (9.5%)	37 (25.3%)
Dyspnea	94 (15.3%)	89 (24.7%)	1(1.1%)	2 (9.5%)	2 (1.4%)
Bronchiolitis	131 (21.3%)	123 (34.1%)	1 (1.1%)	1 (4.8%)	6 (4.1%)
Fever days	1.0 (1.0–1.0)	1.0 (1.0–1.0)	1.0 (1.0–1.0)	1.0 (1.0–1.0)	1.0 (1.0–1.0)

### Laboratory details of study cohort

The details and results about PCR, PCT, urinalysis and lumbar puncture in different diagnostic groups are presented in supplementary materials*.* Regarding inflammatory markers, CRP was measured in 416 patients (67.6%) and repeated within 48–72 h from the first assessment in 140 (22.7%) infants; PCT was measured in 257 patients (41.8%) and repeated in 47 of them (18%). Urinalysis was performed in 318 patients (52.2%) and was suggestive of infection in 96 cases (29.7%). Lumbar puncture was performed in 42 patients (6.8%) for CSF chemical and microbiological analysis. A nasopharyngeal swab for the detection of respiratory viruses was collected in 314 patients (51.1%) with positive detection in 201 cases (63.2%).

### Viral infections

Analysing the association between CRP levels and viral infection, the odds ratio (OR) for CRP was 0.974 (95% CI: 0.964–0.984, p < 0.001), indicating that higher CRP levels were associated with a lower probability of a viral infection. CRP exhibited moderate sensitivity (71.4%) but low specificity (45.2%), leading to a higher false positive rate. Using a cutoff of 5.15 mg/L, CRP exhibited low specificity (33%) in discriminating viral infections, with an AUC value of 0.44.

Increased PCT levels, with an OR of 0.494 (95% CI: 0.311–0.785, *p* = 0.003), were also negatively associated with viral infection. Regarding the diagnostic performance of PCT for identifying viral infection, it demonstrated high sensitivity (92.7%) but low specificity (38.4%). PCT with a cut-off of 0.115 ng/ml reached an AUC value of 0.47.

A logistic regression analysis was conducted to evaluate the role of WBC and ANC in identifying viral infection. WBC was inversely associated with viral infection (OR 0.911, 95% CI: 0.873–0.952, *p* < 0.001), indicating that lower WBC levels were more predictive of viral infections. With a cutoff of 8.775 × 10⁹/L, WBC showed a sensitivity of 52% and a specificity of 37% with an AUC of 0.44. ANC was also inversely associated with viral infections (OR 0.831, 95% CI: 0.772–0.894, *p* < 0.001). Using a cutoff of 2.94 × 10⁹/L, ANC exhibited a sensitivity of 58% and a specificity of 30% with an AUC of 0.44.

### Urinary tract infection

Regarding UTI, the OR for CRP was 1.025 (95% CI: 1.017–1.032, p < 0.001), indicating that higher CRP levels were significantly associated with an increased likelihood of UTI. In our population study CRP demonstrated high specificity but low sensitivity. Using a cut-off of 21.65 mg/L, CRP showed moderate discriminative power (AUC = 0.75) in differentiating UTI.

The OR for PCT was 1.007 (95% CI: 0.97–1.046, *p* = 0.7), suggesting no significant association between PCT levels and UTIs occurrence. Using a cutpoint of 0.185 ng/mL, PCT exhibited moderate discriminative ability (AUC = 0.65).

The comparison of ROC curves for CRP and PCT (*p* = 0.18) suggested no statistically significant difference between the two markers in detecting UTIs. When these biomarkers are associated with a urine analysis, their accuracy improve, but remain similar if using PCT or CRP alone. The details about ROC curves are presented in supplementary materials.

Using a logistic regression analysis, WBC was significantly associated with UTIs (OR 1.187, 95% CI: 1.127–1.249, *p* < 0.001). With a cutoff of 10.88 × 10⁹/L WBC showed a sensitivity of 72% and a specificity of 69%, with an AUC of 0.7. Neutrophil count was also significantly associated with UTIs (OR 1.316, 95% CI: 1.216–1.424, *p* < 0.001). Using a cutoff of 5.505 × 10⁹/L, ANC reached a sensitivity of 70% and a specificity of 79%, with an AUC of 0.74.

### Serious bacterial infection

The OR for CRP was 1.017 (95% CI: 1.008–1.026, *p* < 0.001), suggesting a statistically significant correlation between elevated CRP levels and the occurrence of SBI. CRP with a cutoff value of 12.6 mg/L had a sensitivity of 67% and a specificity of 61%, with an area under the curve (AUC) of 0.64.

The OR for PCT was 1.130 (95% CI: 1.062–1.203, *p* < 0.001), demonstrating a stronger correlation between increased PCT levels and SBI. PCT with a cutoff of 0.78 ng/ml had a sensitivity of 67% and a specificity of 83%, showing a better discriminative power (AUC = 0.75) compared to CRP (AUC = 0.64). The ROC comparison (*p* = 0.13) suggested no statistically significant difference between CRP and PCT in predicting SBI. The details about ROC curves are presented in supplementary materials.

WBC was not significantly associated with SBI (OR 0.995, 95% CI: 0.913–1.084, *p* = 0.905). With a cutoff of 10.235 × 10⁹/L, WBC showed a sensitivity of 38% and a specificity of 54%, with an AUC 0.46, indicating poor discriminative power.

ANC was also not significantly associated with SBI (OR 0.999, 95% CI: 0.991–1.008, *p* = 0.873). The optimal cutpoint was 4.475 × 10⁹/L, with a sensitivity of 57% and a specificity of 61%, with an AUC of 0.59, showing a slightly better discriminative power than WBC.

### Serious bacterial infections (SBIs)

Analysing the population study that had a UTI with bacteraemia (and therefore a SBI), the odds ratio (OR) for CRP was 1.035 (95% CI: 1.025–1.045, *p* < 0.001), demonstrating a strong and statistically significant association between elevated CRP levels and SBI. CRP with a cutpoint of 21.65 mg/L had a sensitivity of 64% and a specificity of 84%, with an AUC of 0.74, showing a moderate discriminative power in distinguishing between cases with and without serious infections.

**Fig. 1:**
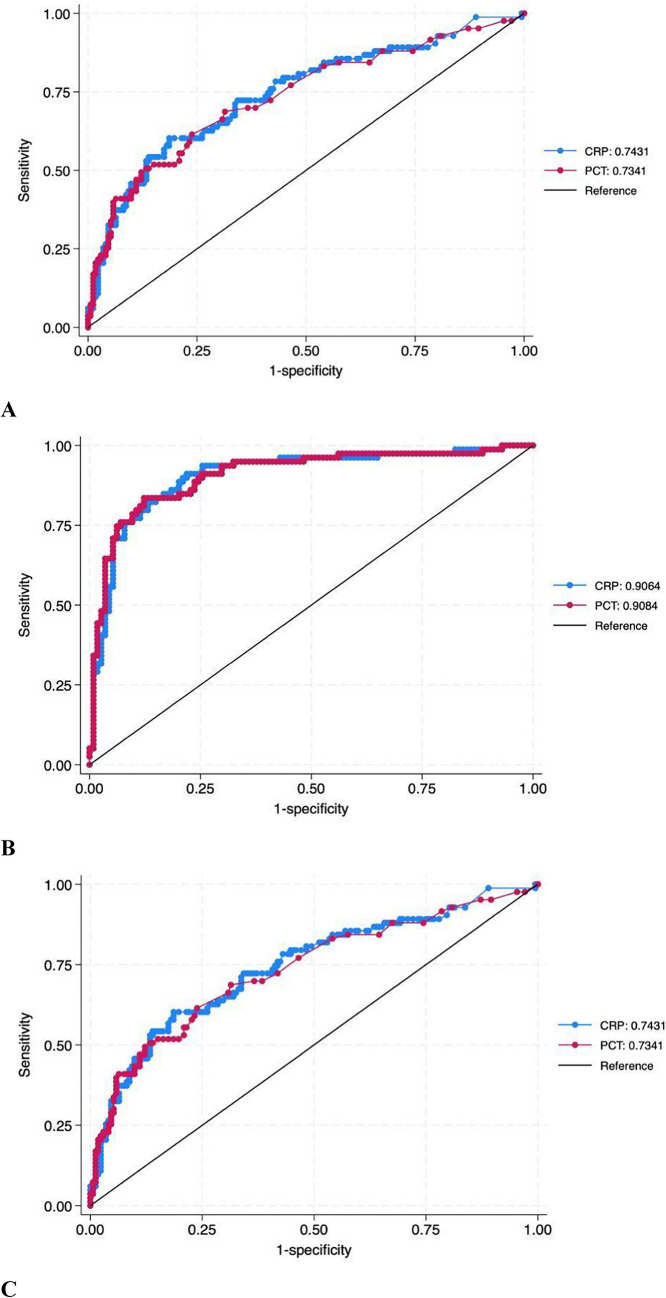
Comparison of ROC curves for CRP and PCT alone (**A**) or with urine analyses (**B**), or using both CRP and PCT (**C**), in discriminating UTIs and SBIs

The OR for PCT was 1.162 (95% CI: 1.054–1.280, *p* = 0.002), demonstrating a significant correlation between elevated PCT levels and UTI with bacteraemia. PCT showed a moderate discriminative power, too, with an AUC of 0.73. The comparison between CRP and PCT ROC curves yielded *p* = 0.80, suggesting no statistically significant difference in their predictive capabilities for UTI with bacteraemia. (Fig. 1A*).* Accuracies strongly improved when CRP or PCT were associated with urinalysis (Fig. 1B*),* while simply using both CRP and PCT did not improve accuracy* (*Fig. 1C*).*

### Multivariate models

We analysed multiple factors for their association with viral infections. Age was found to be a significant predictor, with a slight but statistically significant increase in risk per day (OR 1.021, 95% CI: 1.008–1.035, *p* = 0.002). Failure to thrive was associated with a more than twofold increased likelihood of viral infection (OR 2.189, 95% CI: 1.169–4.097, *p* = 0.014). Among clinical symptoms, nasal congestion (OR 3.237, 95% CI: 1.794–5.840, *p* < 0.001) and dyspnoea (OR 4.584, 95% CI: 1.235–17.012, *p* = 0.023) were strongly associated with viral infections. Conversely, CRP levels demonstrated a weak but significant inverse association (OR 0.98, 95% CI: 0.962–0.999, *p* = 0.036), suggesting that lower CRP values may be indicative of viral infection. Moreover, a positive urinalysis was strongly associated with a lower probability of viral infection (OR 0.098, 95% CI: 0.034–0.283, *p* < 0.001).

A logistic regression model incorporating PCT was used to evaluate factors associated with viral infection. Age remained a significant predictor, with a slight but significant increase in risk per day (OR 1.024, 95% CI: 1.008–1.041, *p* = 0.004). Nasal congestion (OR 2.382, 95% CI: 1.115–5.090, *p* = 0.025) and dyspnoea (OR 7.205, 95% CI: 1.239–41.916, *p* = 0.028) were strongly associated with viral infections. Failure to thrive (OR 1.594, 95% CI: 0.682–3.727, *p* = 0.282) and PCT levels (OR 0.665, 95% CI: 0.32–1.382, *p* = 0.274) did not reach statistical significance in this model. However, a positive urinalysis remained a strong negative predictor of viral infection (OR 0.069, 95% CI: 0.021–0.225, *p* < 0.001). The ROC curve comparison for PCR and PCT resulted in *p* = 0.51, indicating no statistically significant difference between the compared ROC curves.

The details about ROC curves are presented in supplementary materials.

In a multivariate model including white blood cells count and urinalysis, CRP levels were not significantly associated with UTIs (OR 0.998, 95% CI: 0.988–1.007, *p* = 0.622). Conversely, WBC count showed a significant association with UTIs (OR 1.103, 95% CI: 1.039–1.170, *p* = 0.001) and Positive urine chemical test was a strong predictor of UTIs (OR 26.750, 95% CI: 11.890–60.181, *p* < 0.001). A logistic regression analysis was conducted to assess the association of procalcitonin (PCT), white blood cell (WBC) count, and urinalysis with urinary tract infections (UTIs) too. PCT levels were inversely associated with UTIs (OR 0.918, 95% CI: 0.844–0.999, *p* = 0.047). WBC count was positively associated with UTIs (OR 1.086, 95% CI: 1.006–1.173, *p* = 0.035), indicating that an elevated WBC count correlates with an increased likelihood of UTIs. Positive urinalysis was the strongest predictor of UTIs (OR 35.507, 95% CI: 13.776–91.521, *p* < 0.001). The ROC curve comparison between CRP and PCT (*p* = 0.73) showed no statistically significant difference in their discriminative ability for UTIs.

In the logistic regression model, CRP levels were significantly associated with serious bacterial infections (SBIs) (OR 1.014, 95% CI: 1.004–1.024, *p* = 0.007), indicating that higher CRP values correlate with an increased risk of SBI. Age showed a significant inverse association (OR 0.95, 95% CI: 0.923–0.979, *p* = 0.001), suggesting that younger patients were at a higher risk. Furthermore, positive urine chemical tests were identified as a strong predictor of SBIs (OR 3.672, 95% CI: 1.129–11.940, *p* = 0.031). A multivariate logistic regression model was used to evaluate the diagnostic role of Procalcitonin (PCT) for identifying serious bacterial infections (SBIs), too. Age was inversely associated with SBIs (OR 0.927, 95% CI: 0.888–0.969, *p* = 0.001), suggesting that younger children were at higher risk.

PCT levels were significantly associated with SBIs (OR 1.113, 95% CI: 1.050–1.181, *p* < 0.001), confirming its role as a marker of bacterial infections. Positive urine chemical tests didn’t reach statistical significance in this model (OR 3.791, 95% CI: 0.848–16.940, *p* = 0.081).

The discriminative performance of PCT was not significantly superior to that of CRP (*p* = 0.23), suggesting that both biomarkers have comparable diagnostic accuracy for SBIs.

A logistic regression analysis was conducted to analyse the association of age, positive maternal vaginal swab, CRP, neutrophil count, urine chemical test and the occurrence of Serious Bacterial Infections (SBIs) and Urinary Tract Infections (UTIs). Age was not significantly associated with serious UTI (OR 0.982, 95% CI: 0.964–1.000, *p* = 0.054). A positive vaginal swab was significantly associated with an increased likelihood of SBIs and UTIs (OR 4.745, 95% CI: 1.340–16.810, *p* = 0.016). CRP levels did not significantly correlate with the presence of SBIs and UTIs (OR 1.009, 95% CI: 0.997–1.021, *p* = 0.141). Neutrophil count was positively associated with SBIs and UTIs (OR 1.148, 95% CI: 1.042–1.265, *p* = 0.005). A positive urine chemical test was strongly associated with SBIs and UTIs (OR 28.777, 95% CI: 11.993–69.049, *p* < 0.001).

When analyzing PCT levels, the following results were noted: No significant effect of age on SBIs and UTIs was observed (OR 0.992, 95% CI: 0.971–1.013, *p* = 0.453); both a positive vaginal swab (OR 3.303, 95% CI: 0.833–13.097, *p* = 0.089) and PCT levels (OR 1.037, 95% CI: 0.974–1.104, *p* = 0.258) did not significantly correlate with SBIs and UTIs. No significant association between neutrophil count and SBIs and UTIS was found (OR 1.095, 95% CI: 0.98–1.224, *p* = 0.109). Positive urine chemical tests were strongly predictive of SBIs and UTIs (OR 38.602, 95% CI: 13.897–107.228, *p* < 0.001).

**Fig. 2:**
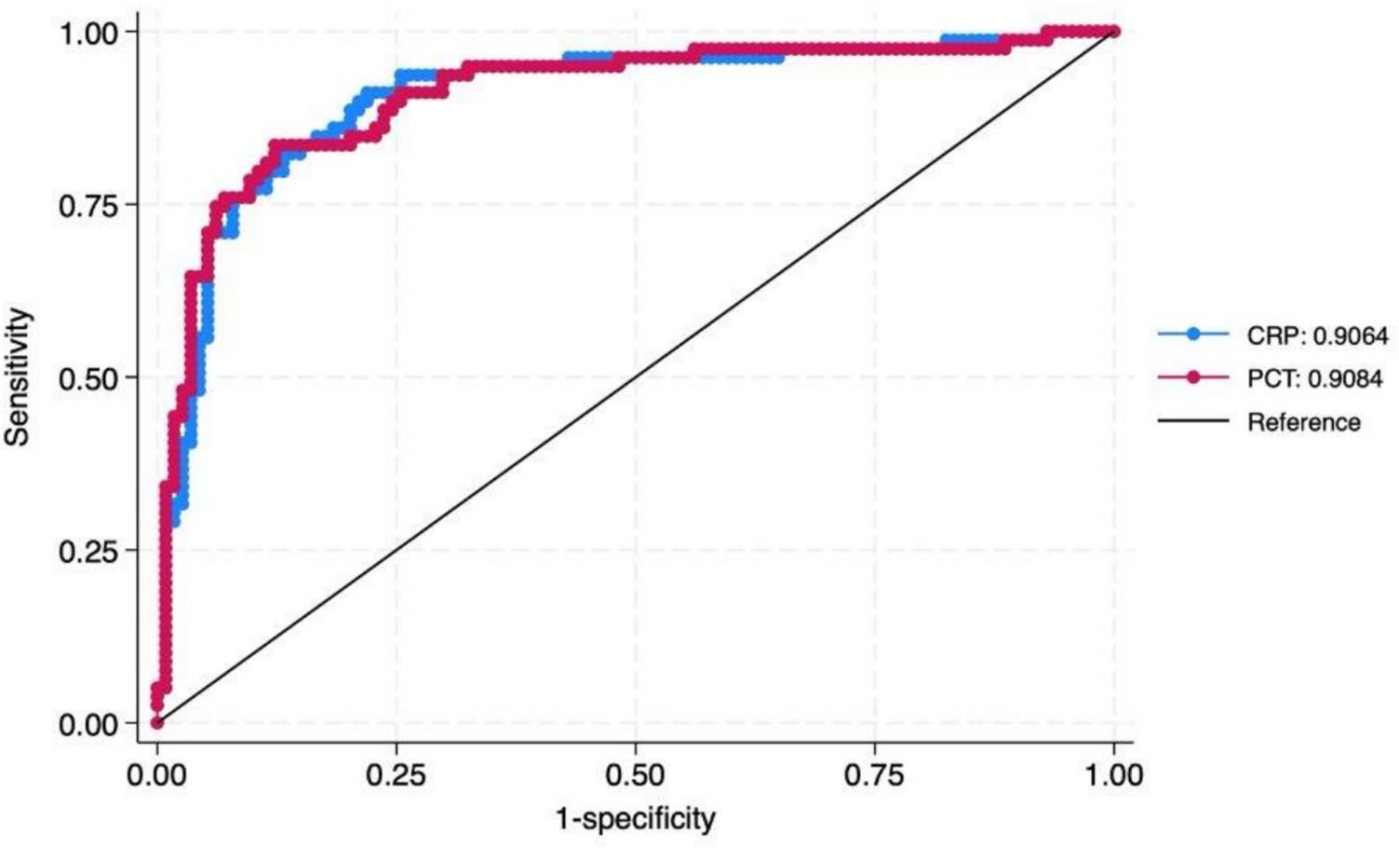
Comparison of ROC curves for CRP and PCT with clinical factors and urine chemical test in discriminating SBIs and UTIs

The comparison of CRP and PCT performance via ROC analysis yielded a *p*-value of 0.77, indicating a moderate discriminative ability of both markers in identifying serious UTIs. (Fig. 2).

All detailed results are also presented in tables in the supplementary material.

### Antibiotic therapy

A total of 212 infants (34.5%) received antibiotic therapy, of which 192 (90.6%) were administered intravenously and 20 (9.4%) received oral antibiotics. The median duration of antibiotic therapy was 5 days. In 64 (10.4%) infants, the intravenous antibiotic therapy was switched to oral administration during the course of treatment. (Table 2).

**Table 2:** Details about antibiotic therapy

	Study population (*N* = 615)	Viral infection (*N* = 361)	Urinary tract infection (*N* = 87)	Serious bacterial infection (*N* = 21)
Atb done	*212 (34.5%)*	61 (16.9%)	84 (96.6%)	21 (100.0%)
Atb route administration				
EV	192 (90.6%)	54 (88.5%)	77 (91.7%)	20 (95.2%)
ORAL	20 (9.4%)	*7 (11.5%)*	*7 (8.3%)*	*1 (4.8%)*
Atb days	5.0 (3.0–7.0)	4.0 (3.0–7.0)	7.0 (4.0–7.0)	7.0 (3.0–11.0)
Shift to oral atb	64 (30.2%)	22 (39.3%)	31 (39.7%)	5 (23.8%)

All detailed results are also presented in tables in the supplementary material.

## Discussion

Despite the diagnostic advances made in recent decades, the management of well appearing young febrile infants remains challenging. Due to the high risk of serious bacterial infections in this age group and the unfavourable outcomes associated with sepsis and meningitis, a cautious approach has been recommended, including invasive diagnostic procedures, such a lumbar puncture, hospitalisation and an immediate start of parenteral antibiotic therapy. Several factors make it necessary to constantly update the approach to this category of patients: changes in the epidemiology of bacterial infection in neonates and infants, such as the reduction of infections caused by *Streptococcus agalactiae* and *Listeria monocytogenes*, and the predominance of infections caused by *Escherichia coli*, [3] improvement in prenatal *Streptococcus agalactiae* screening and in management of intrapartum antibiotic therapy and immunization strategies related to the use of conjugate vaccines. Biomarkers such as procalcitonin, C-reactive protein and white blood cells count are increasingly used to support decision-making as part of a stepwise assessment. One of the aims of our study was to elaborate a strategy that focuses on identifying a lower-risk group, minimizing the need for invasive tests, intravenous antibiotics, and hospital admissions. In our study, conducted on a population of 615 infants aged between 0 and 90 days, we analysed the diagnostic power of CRP, PCT, WBC and ANC in differentiating between viral infections, UTI and SBI, both individually and in combination with other clinical and laboratory data. In patients diagnosed with viral infections, both PCR and PCT demonstrated a low discriminating power, with an AUC value of 0.44 and 0.47, respectively. The diagnostic utility of blood inflammatory markers increases when clinical findings are considered alongside a urinalysis indicative of urinary tract infection. Both PCR and PCT demonstrated a better performance, with an AUC value of 0.86 and 0.87, respectively, with the ROC curve comparison showing no statistically significant difference between PCR and PCT performances. Since these biomarkers alone do not effectively differentiate between viral and bacterial infections, the combination of clinical symptoms and urinalysis should guide the decision-making process, helping in preventing the inappropriate use of antibiotics in this patient population. In our cohort, in fact, only a minority of patients classified with viral infections received antibiotics (16%), suggesting that a similar approach may be appropriate in sparing unnecessary use of antibiotic therapy.

In our study, 14% of young febrile infants received a diagnosis of urinary tract infection; in this population, using an optimal cut-off of 21.65 mg/L, CRP showed moderate discriminative power (AUC = 0.75) in differentiating UTI. Furthermore, it demonstrated a better performance than PCT, which using a cutpoint of 0.185 ng/mL reached an AUC of 0.65. Both PCR and PCT exhibited better discriminatory power in a model including a positive urinalysis and complete WBC. This approach, based on the combination of laboratory tests, may prove useful for clinicians in promptly identifying patients with urinary tract infections, in whom further invasive diagnostic procedures, such as lumbar puncture, could potentially be avoided.

For SBI, CRP was significantly correlated with an OR of 1.017 (*p* < 0.001), with a cutoff of 12.6 mg/L showing a sensitivity of 67% and specificity of 61% (AUC = 0.64). PCT showed a stronger correlation with SBI (OR 1.130, *p* < 0.001) and a better discriminative power (AUC = 0.75) compared to CRP (AUC = 0.64), with a cutoff of 0.78 ng/mL yielding a sensitivity of 67% and specificity of 83%. ROC analysis suggested no significant difference between CRP and PCT in predicting SBI (*p* = 0.13). These results are in line with those reported in a systematic review and meta-analysis [7] which demonstrated that a cutoff of 0.5 ng/mL for procalcitonin was superior to C-reactive protein, with a cutoff of 20 mg/L, in detecting invasive bacterial infection in young febrile infants.

Our data confirmed poor accuracy of WBC and neutrophil count in discriminating young febrile infants with serious bacterial infections, in line with the results reported in available literature. [8–10]

In our multivariate model, when taking age and urinalysis into consideration, positive urinalysis was a strong predictor of SBI when using CRP, though they did not reach statistical significance with PCT. In the group of patients with UTI with bacteraemia, CRP was significantly associated with these infections, showing an OR of 1.035 (p < 0.001). At a cutoff of 21.65 mg/L, CRP demonstrated a sensitivity of 64%, specificity of 84%, and an AUC of 0.74, indicating moderate discriminative power for identifying serious infections. PCT also showed a significant correlation with SBI and UTI, with an OR of 1.162 (p = 0.002), and demonstrated a moderate discriminative power (AUC = 0.73). The comparison of ROC curves for CRP and PCT yielded a p-value of 0.80, suggesting no significant difference in their ability to predict SBI and UTI.

In a single centre study conducted on 2032 young febrile infants, Marom et al. [11] reported that the combination of CRP and PCT could predict invasive bacterial infection with high specificity (85.6%) in patients older than 21 days, suggesting that an approach based on the evaluation of both inflammatory markers could help decision-making on further management. Unfortunately, we could not analyse the discriminative power of both inflammatory markers since they were only available in a smaller percentage of our population.

Overall, our findings suggest that single biomarkers are less useful in discriminating between viral and bacterial infection than a combination of biomarkers and clinical findings. These data are mostly in line with the PECARN step-by-step approach [12, 13], which used three laboratory test results (the urinalysis, the ANC and procalcitonin serum levels) to identify infants at low risk of SBIs and with the Step-by-step approach proposed by a European group of paediatric emergency physicians [14] which evaluated sequentially the general appearance of the infant, the age and the results of urinalysis and blook inflammatory biomarkers to identify a low-risk group in which lumbar puncture and antibiotic therapy can be safely avoided.

although we did not find any clinically relevant differences between CRP and PCT, suggesting that using both these markers routinely may be expensive and unnecessary. Importantly, our study also suggests that an approach based on clinical findings plus one biomarker was reassuring for most clinicians that did not proceed in further investigation in most cases classified as viral infections. Of note, during follow-up, none of the patients received a different diagnosis, as we have a dedicated outpatient unit. As such, the accuracies of our models are realistic and applicable in the real life.

Notably, a significant proportion of our study population was transitioned from intravenous to oral antibiotics, in line with more recent literature from adults and older children showing that both routes of administration may be equally effective (https://www.rch.org.au/uploadedFiles/Main/Content/clinicalguide/guideline_index/IV%20to%20oral%20switch.pdf). Despite the level of evidence for young infants being lower compared with adults [15, 16], this practice is being increasingly adopted in our centre without major complications.

The main limitation of our study is represented by its observational retrospective nature, although overcome by a large cohort and the implementation of multiple parametric models.

In conclusion, we found that febrile infants should be managed assessing both clinical and laboratory parameters, in this way supporting the clinician in accurately recognizing low risk infants with viral infections. A similar approach, if prospectively validated, may help sparing admissions and antibiotics in most febrile infants and, according to our data, could be feasible to Italian children. Our study’s retrospective design does not allow us to determine whether lumbar puncture is always necessary or whether a shorter course of intravenous antibiotics would affect patient outcomes; however our findings may lay the groundwork for a prospective study (potentially a clinical trial) aimed at identifying low-risk patients who may not require LP or prolonged antibiotic therapy. This is especially relevant given that LP is an invasive procedure and that increasing evidence suggests early antibiotic exposure in neonates may have long-term health implications.

## Supplementary Information

Below is the link to the electronic supplementary material.

Supplementary file1 (DOCX 261 KB)

## Data Availability

No datasets were generated or analysed during the current study.
