# Supplementary material for: Diagnostic multiparametric models and antibiotic practices in febrile infants younger than 90 days
Source: Eur J Pediatr. 2025 Oct 9;184(11):669. doi: 10.1007/s00431-025-06528-4 (PMC12511232; doi:10.1007/s00431-025-06528-4)
Supplement: Supplementary file 1 — Supplementary file1 (DOCX 261 KB) [file 431_2025_6528_MOESM1_ESM.docx]

**Supplementary materials**

|  | ***Study population (N=615)*** |
| --- | --- |
| ***PCT done*** | 257 (41.8%) |
| ***PCT value*** | 0.2 (0.1-0.4) |
| ***Second PCT done*** | 47 (18.0%) |
| ***Second PCT value*** | 0.3 (0.1-1.1) |
| ***CRP done*** | 416 (67.6%) |
| ***CRP value*** | 8.6 (2.2-24.9) |
| ***Second CRP done*** | 140 (33.7%) |
| ***Second CRP value*** | 9.9 (1.2-26.6) |
| ***Both first CRP and PCT negative*** | 73 (30.3% |
| ***WBC done*** | 404 (65.7%) |
| ***WBC*** | 9.8 (6.9-12.9) |
| ***Neutrophils count*** | 3.7 (2.4-6.4) |
| ***Urinalysis done*** | 318 (52.2%) |
| ***Urinalysis suggestive of infection*** | 96 (29.7%) |
| ***Presence of esterase*** | 98 (30.7%) |
| ***Presence of nitrites*** | 31 (9.7%) |
| ***Lumbar puncture done*** | 42 (6.8%) |
| ***CSF glucose*** | 58.5 (53.5-65.5) |
| ***CSF cells*** | 0.0 (0.0-3.5) |
| ***CSF proteins*** | 48.5 (40.0-67.0) |

*Table 1 details and results of the laboratory tests in the study population (Data are presented as median (IQR) for continuous measures, and n (%) for categorical measures.)*

|  | ***Viral infection (N=361)*** | ***Urinary tract infection (N=87)*** | ***Serious bacterial infection (N=21)*** | ***Undefined diagnosis (N=146)*** |
| --- | --- | --- | --- | --- |
| ***PCT done*** | *123 (34.1%)* | *65 (74.7%)* | *18 (85.7%)* | *51 (34.9%)* |
| ***PCT value*** | *0.1 (0.1-0.2)* | *0.2 (0.1-1.2)* | *1.2 (0.2-21.3)* | *0.1 (0.1-0.4)* |
| ***Second PCT done*** | *19 (15.1%)* | *11 (16.4%)* | *10 (55.6%)* | *7 (14.0%)* |
| ***Second PCT value*** | *0.2 (0.1-0.3)* | *0.5 (0.3-2.8)* | *1.7 (0.1-25.0)* | *0.4 (0.3-1.1)* |
| ***CRP done*** | *199 (55.1%)* | *83 (95.4%)* | *21 (100.0%)* | *113 (77.4%)* |
| ***CRP value*** | *6.3 (2.0-16.2)* | *38.7 (13.0-68.2)* | *14.0 (9.2-68.3)* | *4.8 (0.7-13.3)* |
| ***Second CRP done*** | *43 (21.6%)* | *44 (53.0%)* | *18 (85.7%)* | *35 (31.0%)* |
| ***Second CRP value*** | *7.8 (0.5-14.9)* | *13.1 (3.5-39.2)* | *44.0 (5.0-78.8)* | *7.1 (0.5-16.1)* |
| ***Both first CRP and PCT negative*** | *43 (38.7%)* | *8 (13.1%)* | *2 (10.5%)* | *20 (40.0%)* |
| ***WBC done*** | *189 (52.4%)* | *81 (93.1%)* | *21 (100.0%)* | *113 (77.4%)* |
| ***WBC*** | *8.9 (6.6-11.3)* | *14.3 (10.4-19.2)* | *7.5 (5.9-12.1)* | *9.6 (6.6-12.2)* |
| ***Neutrophils count*** | *3.2 (2.2-4.6)* | *7.3 (4.7-10.5)* | *4.5 (3.1-7.5)* | *3.5 (2.4-5.9)* |
| ***Urinalysis done*** | *117 (33.0%)* | *85 (97.7%)* | *20 (95.2%)* | *96 (65.8%)* |
| ***Urinalysis suggestive of infection*** | *6 ( 5.0%)* | *68 (79.1%)* | *10 (50.0%)* | *12 (12.2%)* |
| ***Presence of esterase*** | *7 ( 6.0%)* | *68 (80.0%)* | *10 (50.0%)* | *13 (13.3%)* |
| ***Presence of nitrites*** | *0 ( 0.0%)* | *24 (28.2%)* | *6 (30.0%)* | *1 ( 1.0%)* |
| ***Both esterase and nitrites positive*** | *0 ( 0.0%)* | *22 (25.9%)* | *6 (30.0%)* | *0 ( 0.0%)* |
| ***Lumbar puncture done*** | *9 ( 2.5%)* | *12 (13.8%)* | *11 (52.4%)* | *10 ( 6.8%)* |
| ***CSF glucose*** | *63.0 (54.0-67.0)* | *57.0 (51.0-64.0)* | *56.0 (53.0-72.0)* | *60.0 (56.0-72.0)* |
| ***CSF cells*** | *0.0 (0.0-0.0)* | *0.0 (0.0-15.0)* | *2.0 (0.0-9.0)* | *1.0 (0.0-3.0)* |
| ***CSF proteins*** | *40.0 (35.0-47.0)* | *47.0 (41.0-67.0)* | *67.0 (50.0-76.0)* | *56.0 (39.0-59.0)* |

*Table 2 Details and results of the laboratory tests in the  diagnosis groups (Data are presented as median (IQR) for continuous measures, and n (%) for categorical measures.)*

|  |  | **Total** |
| --- | --- | --- |
|  |  |  |
|  |  | N=615 |
|  |  |  |
| Vaginal swab | Negative | 197 (32.0%) |
|  | Positive | 35 ( 5.7%) |
|  | Unknown | 383 (62.3%) |
|  |  |  |
| Microrganism in vaginal swab | Streptococcus Agalactiae | 31 ( 88.5%) |
|  | Gardnerella | 2 ( 5.7%) |
|  | K. Pneumoniae | 1 ( 2.8%) |
|  |  |  |
| Urinocolture positive |  | 105 (41.2%) |
|  |  |  |
| Microrganism detected in the Urinocolture | E. Coli | 79 (75.2%) |
|  | S. Aureus | 1 ( 0.9%) |
|  | Ps. Aer | 4 ( 3.8%) |
|  | E. Faecalis | 18 ( 17.1%) |
|  | K. Pneum | 6 ( 5.7 %) |
|  | K. Oxytoca | 8 ( 7.6 %) |
|  | St. Faecalis | 3 ( 0.5 %) |
|  | Citrob. K. | 6 ( 5.7 %) |
|  | Enterobacter | 4 (3.8%) |
|  | Str. Agal | 1 ( 0.9%) |
|  | Proteus Mir. | 2 ( 1.8%) |
|  | S. Gallolyt | 1 ( 0.9%) |
|  | Str. Agal | 1 ( 0.9%) |
|  |  |  |
| Blood colture done |  | 203 (33.0%) |
|  |  |  |
| Blood colture positive |  | 17 (8.3%) |
|  |  |  |
| Microrganism in blood colture | E. Coli | 10 ( 58.8%) |
|  | S. Aureus | 2 ( 11.7%) |
|  | Ps. Aer | 1 ( 5.8%) |
|  | Str. Agal | 3 ( 17.6%) |
|  | S. Hominis | 1 ( 5.8%) |
|  |  |  |
| Nasopharyngeal swab done |  | 314 (51.1%) |
|  |  |  |
| Nasopharyngeal swab positive |  | 201 (63.2%) |
|  |  |  |
| Microrganism detected at Nasopharyngeal swab | Rhino/Enterovirus | 64 ( 31.8%) |
|  | RSV | 54 ( 26.8%) |
|  | Covid | 74 (36.8%) |
|  | Influenza B | 3 ( 1.5%) |
|  | Parainfluenzae | 7 ( 3.5%) |
|  | Metapn. | 2 ( 0.9%) |
|  | Coronavirus | 5 ( 2.5%) |
|  | Influenza A | 9 ( 4.5%) |
|  | Adenov | 5 ( 2.5%) |
|  | H. Influenzae | 1 ( 0.5%) |
|  |  |  |
|  |  |  |
| CSF colture  positive |  | 9 (21.4%) |
|  |  |  |
| Microrganism detected in CSF | E. Coli | 2 ( 22.2%) |
|  | Str. Agal | 1 ( 11.1%) |
|  | S. Epiderm | 1 ( 11.1%) |
|  | Rhino/Enterovirus | 3 ( 33.3%) |
|  | Parechov | 3 ( 33.3%) |

**Table 3. Microbiological data in the study population (Data are presented as median (IQR) for continuous measures, and n (%) for categorical measures.)**

|  |  | **Total** |
| --- | --- | --- |
|  |  |  |
|  |  | N=615 |
|  |  |  |
| Antibiotic therapy done |  | 212 (34.5%) |
|  |  |  |
| Place of start Antibiotic therapy | ED | 60 (28.3%) |
|  | Ward | 145 (68.4%) |
|  | Home | 7 ( 3.3%) |
|  |  |  |
| Name of first Antibiotic used | Ampicillin | 33 (15.5%) |
|  | Ampi-Sulb | 1 ( 0.5%) |
|  | Amoxicillin | 2 ( 0.9%) |
|  | Amoxi-Clav | 47 (22.1%) |
|  | Amikacin | 82 (38.5%) |
|  | Gentamicin | 11 ( 5.2%) |
|  | Ceftriaxone | 30 (14.1%) |
|  | Oxacillin | 1 ( 0.5%) |
|  | Azithromicin | 2 ( 0.9%) |
|  | Meropenem | 1 ( 0.5%) |
|  | Cefixime | 1 ( 0.5%) |
|  | Cefepime | 1 ( 0.5%) |
|  | Teicoplanin | 1 ( 0.5%) |
|  |  |  |
| IV/ORAL | IV | 192 (90.6%) |
|  | ORAL | 20 ( 9.4%) |
|  |  |  |
| Antibiotic therapy duration (days) |  | 5.0 (3.0-7.0) |
| Second Antibiotic therapy received |  | 145 (23.5%) |
| Name of second Antibiotic therapy | Ampicillin | 77 (53.1%) |
|  | Ampi-Sulb | 1 ( 0.7%) |
|  | Amoxi-Clav | 7 ( 4.8%) |
|  | Amikacin | 23 (15.9%) |
|  | Gentamicin | 12 ( 8.3%) |
|  | Ceftriaxone | 2 ( 1.4%) |
|  | Azithromicin | 2 ( 1.4%) |
|  | Cefixime | 4 ( 2.8%) |
|  | Cefepime | 2 ( 1.4%) |
|  | Teicoplanin | 8 ( 5.5%) |
|  | Ceftazidime | 1 ( 0.7%) |
|  | Cefaclor | 1 ( 0.7%) |
|  | Clarithromicin | 3 ( 2.1%) |
|  | Trimetoprim | 2 ( 1.4%) |
|  |  |  |
| Route of administration | ORAL | 18 (12.4%) |
|  | IV | 127 (87.6%) |
| Second Antibiotic therapy days |  | 5.0 (3.0-7.0) |
| Third Antibiotic therapy received |  | 29(4.7%) |
| Name of third Antibiotic therapy | Ampicillin | 1 ( 3.4%) |
|  | Amoxicillin | 1 ( 3.4%) |
|  | Amoxi-Clav | 11 (37.9%) |
|  | Ceftriaxone | 4 (13.8%) |
|  | Meropenem | 1 ( 3.4%) |
|  | Cefixime | 3 (10.3%) |
|  | Cefepime | 1 ( 3.4%) |
|  | Teicoplanin | 1 ( 3.4%) |
|  | Ceftazidime | 4 (13.8%) |
|  | Cefaclor | 1 ( 3.4%) |
|  | Trimetoprim | 1 ( 3.4%) |
|  |  |  |
| Route of administration of third Antibiotic therapy | ORAL | 12 (41.4%) |
|  | IV | 17 (58.6%) |
|  |  |  |
| Third Antibiotic therapy days |  | 6.0 (5.0-7.0) |
|  |  |  |
| Shift to oral Antibiotic therapy |  | 64 (32.5%) |
|  | No | 133 (67.5%) |

**Table 4. Details about therapy in the population study.**

|  |  | **Viral Infect** | **Urinary tract** | **Serious bacterial** | **Undef** | **p-value** |
| --- | --- | --- | --- | --- | --- | --- |
|  |  |  |  |  |  |  |
|  |  | N=361 | N=87 | N=21 | N=146 |  |
|  |  |  |  |  |  |  |
| Vaginal swab | Negative | 77 (21.3%) | 44 (50.6%) | 12 (57.1%) | 64 (43.8%) | <0.001 |
|  | Positive | 14 ( 3.9%) | 9 (10.3%) | 5 (23.8%) | 7 ( 4.8%) |  |
|  | Unknown | 270 (74.8%) | 34 (39.1%) | 4 (19.0%) | 75 (51.4%) |  |
|  |  |  |  |  |  |  |
| Microrganism in vaginal swab | Str. Agal | 12 (100.0%) | 7 (77.8%) | 5 (100.0%) | 7 (100.0%) | 0.13 |
|  | Gardnerella | 0 ( 0.0%) | 2 (22.2%) | 0 ( 0.0%) | 0 ( 0.0%) |  |
|  | K. Pneum |  |  |  | 1 (100.0%) |  |
|  |  |  |  |  |  |  |
|  |  |  |  |  |  |  |
| Urinocolture positive |  | 10 (12.0%) | 81 (94.2%) | 12 (60.0%) | 2 ( 3.0%) | <0.001 |
|  |  |  |  |  |  |  |
| Microrganism UC | 0 | 1 (10.0%) | 2 ( 2.5%) | 1 ( 8.3%) | 0 ( 0.0%) | <0.001 |
|  | E. Coli | 7 (70.0%) | 62 (76.5%) | 9 (75.0%) | 1 (50.0%) |  |
|  | S. Aureus | 0 ( 0.0%) | 0 ( 0.0%) | 1 (8.3%) | 0 ( 0.0%) |  |
|  | Ps. Aer | 0 ( 0.0%) | 3 ( 3.7%) | 1 (8.3%) | 0 ( 0.0%) |  |
|  | E. Faecalis | 4 (40.0%) | 14 ( 17.3%) | 0 ( 0.0%) | 0 ( 0.0%) |  |
|  | K. Pneum | 1 ( 0.0%) | 1 (1.2%) | 0 ( 0.0%) | 0 ( 0.0%) |  |
|  | K. Oxytoca | 1 ( 0.0%) | 7 ( 8.6%) | 0 ( 0.0%) | 0 ( 0.0%) |  |
|  | St. Faecalis | 1 ( 10.0%) | 1 ( 1.2%) | 0 ( 0.0%) | 1 (50.0%) |  |
|  | Citrob. K. | 1 (10.0%) | 3 ( 3.7%) | 0 ( 0.0%) | 0 ( 0.0%) |  |
|  | Enterobacter | 0 ( 0.0%) | 4 ( 4.9%) | 0 ( 0.0%) | 0 ( 0.0%) |  |
|  | Str. Agal | 0 ( 0.0%) | 1 (1.2%) | 1 (8.3%) | 0 ( 0.0%) |  |
|  | Str. Anginosus | 0 ( 0.0%) | 1 ( 1.2%) | 0 ( 0.0%) |  |  |
|  | Proteus Mir. | 0 ( 0.0%) | 1 ( 1.2%) | 1 (8.3%) |  |  |
|  | S. Gallolyt | 0 ( 0.0%) | 1 ( 1.2%) | 0 ( 0.0%) |  |  |
|  |  |  |  |  |  |  |
|  |  |  |  |  |  |  |
|  |  |  |  |  |  |  |
|  |  |  |  |  |  |  |
| Blood colture done |  | 57 (15.8%) | 72 (82.8%) | 21 (100.0%) | 53 (36.3%) | <0.001 |
|  |  |  |  |  |  |  |
| Blood colture positive |  | 0 ( 0.0%) | 1 ( 1.4%) | 15 (71.4%) | 1 ( 1.9%) | <0.001 |
|  |  |  |  |  |  |  |
| Microrganism in blood colture | E. Coli |  | 0 ( 0.0%) | 9 (60.0%) | 1 (100.0%) | 0.024 |
|  | S. Aureus |  | 0 ( 0.0%) | 2 (13.3%) | 0 ( 0.0%) |  |
|  | Ps. Aer |  | 0 ( 0.0%) | 1 ( 6.7%) | 0 ( 0.0%) |  |
|  | Str. Agal |  | 0 ( 0.0%) | 3 (20.0%) | 0 ( 0.0%) |  |
|  | S. Hominis |  | 1 (100.0%) | 0 ( 0.0%) | 0 ( 0.0%) |  |
|  |  |  |  |  |  |  |
| Nasopharyngeal (NP) swab done |  | 202 (56.0%) | 48 (55.2%) | 13 (61.9%) | 51 (34.9%) | <0.001 |
|  |  |  |  |  |  |  |
| NP swab positive |  | 185 (90.7%) | 11 (22.9%) | 5 (38.5%) | 0 ( 0.0%) | <0.001 |
|  |  |  |  |  |  |  |
| Microrganism NP swab | Rhino/Enterovirus | 52 (28.1%) | 9 (81.8%) | 3 (60.0%) |  | <0.001 |
|  | RSV | 52 (28.1%) | 2 ( 18.2%) | 0 ( 0.0%) |  |  |
|  | Covid | 71 (38.4%) | 2 (18.2%) | 1 (20.0%) |  |  |
|  | Influenza B | 3 ( 1.6%) | 0 ( 0.0%) | 0 ( 0.0%) |  |  |
|  | Parainfluenzae | 7 ( 3.7%) | 0 ( 0.0%) | 0 ( 0.0%) |  |  |
|  | Metapn. | 1 ( 0.5%) | 0 ( 0.0%) | 0 ( 0.0%) |  |  |
|  | Coronavirus | 5 ( 2.7%) | 0 ( 0.0%) | 0 ( 0.0%) |  |  |
|  | Influenza A | 9 ( 4.9%) | 0 ( 0.0%) | 0 ( 0.0%) |  |  |
|  | Adenov | 5 (2.7%) | 0 ( 0.0%) | 0 ( 0.0%) |  |  |
|  | H. Infl | 0 ( 0.0%) | 0 ( 0.0%) | 1 (20.0%) |  |  |
|  | Metapn | 1 ( 0.5%) | 0 ( 0.0%) | 0 ( 0.0%) |  |  |
|  |  |  |  |  |  |  |
|  |  |  |  |  |  |  |
| CSF colture positive |  | 1 (11.1%) | 0 ( 0.0%) | 8 (72.7%) | 0 ( 0.0%) | <0.001 |
|  |  |  |  |  |  |  |
| Microrganism in CSF | E. Coli | 0 ( 0.0%) |  | 2 (25.0%) |  | 0.21 |
|  | Str. Agal | 0 ( 0.0%) |  | 1 (12.5%) |  |  |
|  | S. Epiderm | 0 ( 0.0%) |  | 1 (12.5%) |  |  |
|  | Rhino/Enterovirus | 0 ( 0.0%) |  | 3 (37.5%) |  |  |
|  | Parechov | 2 (100.0%) |  | 1 (12.5%) |  |  |
|  |  |  |  |  |  |  |
| Antibiotic therapy done |  | 61 (16.9%) | 84 (96.6%) | 21 (100.0%) | 46 (31.5%) | <0.001 |
|  |  |  |  |  |  |  |
| Antibiotic therapy where started | ED | 20 (32.8%) | 26 (31.0%) | 5 (23.8%) | 9 (19.6%) | 0.71 |
|  | Ward | 39 (63.9%) | 55 (65.5%) | 16 (76.2%) | 35 (76.1%) |  |
|  | Home | 2 ( 3.3%) | 3 ( 3.6%) | 0 ( 0.0%) | 2 ( 4.3%) |  |
|  |  |  |  |  |  |  |
| Name of First Antibiotic used | Ampicillin | 12 (19.7%) | 12 (14.1%) | 5 (23.8%) | 4 ( 8.7%) | <0.001 |
|  | Ampi-Sulb | 1 ( 1.6%) | 0 ( 0.0%) | 0 ( 0.0%) | 0 ( 0.0%) |  |
|  | Amoxicillin | 1 ( 1.6%) | 0 ( 0.0%) | 0 ( 0.0%) | 1 ( 2.2%) |  |
|  | Amoxi-Clav | 23 (37.7%) | 21 (24.7%) | 1 ( 4.8%) | 2 ( 4.3%) |  |
|  | Amikacin | 13 (21.3%) | 31 (36.5%) | 6 (28.6%) | 32 (69.6%) |  |
|  | Gentamicin | 3 ( 4.9%) | 3 ( 3.5%) | 2 ( 9.5%) | 3 ( 6.5%) |  |
|  | Ceftriaxone | 6 ( 9.8%) | 18 (21.2%) | 3 (14.3%) | 3 ( 6.5%) |  |
|  | Oxacillin | 0 ( 0.0%) | 0 ( 0.0%) | 1 ( 4.8%) | 0 ( 0.0%) |  |
|  | Azithromicin | 2 ( 3.3%) | 0 ( 0.0%) | 0 ( 0.0%) | 0 ( 0.0%) |  |
|  | Meropenem | 0 ( 0.0%) | 0 ( 0.0%) | 1 ( 4.8%) | 0 ( 0.0%) |  |
|  | Cefixime | 0 ( 0.0%) | 0 ( 0.0%) | 0 ( 0.0%) | 1 ( 2.2%) |  |
|  | Cefepime | 0 ( 0.0%) | 0 ( 0.0%) | 1 ( 4.8%) | 0 ( 0.0%) |  |
|  | Teicoplanin | 0 ( 0.0%) | 0 ( 0.0%) | 1 ( 4.8%) | 0 ( 0.0%) |  |
|  |  |  |  |  |  |  |
| route of administration af Antibiotic therapy | IV | 54 (88.5%) | 77 (91.7%) | 20 (95.2%) | 41 (89.1%) | 0.79 |
|  | ORAL | 7 (11.5%) | 7 ( 8.3%) | 1 ( 4.8%) | 5 (10.9%) |  |
|  |  |  |  |  |  |  |
| Antibiotic therapy duration (days) |  | 4.0 (3.0-7.0) | 7.0 (4.0-7.0) | 7.0 (3.0-11.0) | 5.0 (3.0-6.0) | <0.001 |
|  |  |  |  |  |  |  |
| Name of second Antibiotic therapy | Ampicillin | 11 (32.4%) | 26 (50.0%) | 8 (42.1%) | 32 (80.0%) | 0.055 |
|  | Ampi-Sulb | 1 ( 2.9%) | 0 ( 0.0%) | 0 ( 0.0%) | 0 ( 0.0%) |  |
|  | Amoxi-Clav | 3 ( 8.8%) | 3 ( 5.8%) | 1 ( 5.3%) | 0 ( 0.0%) |  |
|  | Amikacin | 7 (20.6%) | 10 (19.2%) | 5 (26.3%) | 1 ( 2.5%) |  |
|  | Gentamicin | 5 (14.7%) | 3 ( 5.8%) | 2 (10.5%) | 2 ( 5.0%) |  |
|  | Ceftriaxone | 0 ( 0.0%) | 1 ( 1.9%) | 0 ( 0.0%) | 1 ( 2.5%) |  |
|  | Azithromicin | 2 ( 5.9%) | 0 ( 0.0%) | 0 ( 0.0%) | 0 ( 0.0%) |  |
|  | Cefixime | 0 ( 0.0%) | 3 ( 5.8%) | 0 ( 0.0%) | 1 ( 2.5%) |  |
|  | Cefepime | 0 ( 0.0%) | 1 ( 1.9%) | 1 ( 5.3%) | 0 ( 0.0%) |  |
|  | Teicoplanin | 3 ( 8.8%) | 3 ( 5.8%) | 1 ( 5.3%) | 1 ( 2.5%) |  |
|  | Ceftazidime | 0 ( 0.0%) | 0 ( 0.0%) | 0 ( 0.0%) | 1 ( 2.5%) |  |
|  | Cefaclor | 0 ( 0.0%) | 0 ( 0.0%) | 0 ( 0.0%) | 1 ( 2.5%) |  |
|  | Clarithromicin | 2 ( 5.9%) | 0 ( 0.0%) | 1 ( 5.3%) | 0 ( 0.0%) |  |
|  | Trimetoprim | 0 ( 0.0%) | 2 ( 3.8%) | 0 ( 0.0%) | 0 ( 0.0%) |  |
|  |  |  |  |  |  |  |
| second Antibiotic therapy administered orally |  | 7 (20.6%) | 8 (15.4%) | 2 (10.5%) | 1 ( 2.5%) | 0.1 |
|  |  |  |  |  |  |  |
| second Antibiotic therapy duration (days) |  | 3.0 (3.0-5.0) | 5.0 (3.0-7.0) | 5.0 (3.0-10.0) | 4.5 (3.0-5.5) | 0.042 |
|  |  |  |  |  |  |  |
| Third Antibiotic therapy | Ampicillin | 0 ( 0.0%) | 1 ( 6.2%) | 0 ( 0.0%) | 0 ( 0.0%) | 0.52 |
|  | Amoxicillin | 0 ( 0.0%) | 1 ( 6.2%) | 0 ( 0.0%) | 0 ( 0.0%) |  |
|  | Amoxi-Clav | 2 (66.7%) | 5 (31.2%) | 2 (33.3%) | 2 (50.0%) |  |
|  | Ceftriaxone | 0 ( 0.0%) | 2 (12.5%) | 1 (16.7%) | 1 (25.0%) |  |
|  | Meropenem | 0 ( 0.0%) | 0 ( 0.0%) | 1 (16.7%) | 0 ( 0.0%) |  |
|  | Cefixime | 0 ( 0.0%) | 3 (18.8%) | 0 ( 0.0%) | 0 ( 0.0%) |  |
|  | Cefepime | 0 ( 0.0%) | 1 ( 6.2%) | 0 ( 0.0%) | 0 ( 0.0%) |  |
|  | Teicoplanin | 1 (33.3%) | 0 ( 0.0%) | 0 ( 0.0%) | 0 ( 0.0%) |  |
|  | Ceftazidime | 0 ( 0.0%) | 2 (12.5%) | 2 (33.3%) | 0 ( 0.0%) |  |
|  | Cefaclor | 0 ( 0.0%) | 0 ( 0.0%) | 0 ( 0.0%) | 1 (25.0%) |  |
|  | Trimetoprim | 0 ( 0.0%) | 1 ( 6.2%) | 0 ( 0.0%) | 0 ( 0.0%) |  |
|  |  |  |  |  |  |  |
| third Antibiotic therapy administered orally |  | 1 (33.3%) | 8 (50.0%) | 2 (33.3%) | 1 (25.0%) | 0.76 |
|  |  |  |  |  |  |  |
| Third Antibiotic therapy duration (days) |  | 5.0 (3.0-5.0) | 6.0 (4.0-7.0) | 6.5 (5.0-10.0) | 6.0 (4.0-10.5) | 0.37 |
|  |  |  |  |  |  |  |
| Shift to oral Antibiotic therapy |  | 22 (39.3%) | 31 (39.7%) | 5 (23.8%) | 6 (14.3%) | 0.018 |

**Table 4. Microbiological data and details about therapy in the different diagnosis groups.**

**Viral infections**

|  | **CRP (mg/L)** | **PCT (ng/ml)** | **WBC (x10^9/L)** | **Neutrophil count (x10^9/L)** |
| --- | --- | --- | --- | --- |
| **OR** | 0.974 (95% CI: 0.964 – 0.984, p < 0.001) | 0.494 (95% CI: 0.311 – 0.785, p = 0.003) | 0.911 (95% CI: 0.873 – 0.952, p=0) | 0.831 (95% CI: 0.772 – 0.894, p=0) |
| **Sensitivity** | 71.36% | 92.74% | 50.27% |  |
| **Specificity** | 45.16% | 38.35% | 62.62% |  |
| **Positive predictive value** | 54.41% | 58.38% | 54.02% |  |
| **Negative predictive value** | 63.23% | 85.00% | 59.03% |  |
| **Empirical optimal cutpoint:** | 5.15 | 0.115 | 8.775 | 2.94 |
| **Sensitivity at cutpoint** | 56% | 0.68 | 0.52 | 0.58 |
| **Specificity at cutpoint** | 33% | 0.27 | 0.37 | 0.3 |
| **Area under ROC curve at cutpoint** | 0.44 | 0.47 | 0.44 | 0.44 |

**Urinary tract infections**

|  | **CRP (mg/L)** | **PCT (ng/ml)** | **WBC (x10^9/L)** | **Neutrophil count (x10^9/L)** |
| --- | --- | --- | --- | --- |
| **OR** | 1.025(95% CI: 1,017 – 1.032, p < 0.001) | 1.007(95% CI: 0.97 – 1.046, p < 0.001) | 1.187(95% CI: 1.127 – 1.249 p < 0.001) | 1.316 (95% CI: 1.216 – 1.424, p < 0.001) |
| **Sensitivity** | 18.07% | 0 | 23.46% | 23.75% |
| **Specificity** | 97.0% | 100% | 96.88% | 97.14% |
| **Positive predictive value** | 60% |  | 65.52% | 67.86% |
| **Negative predictive value** | 82.6% | 74.71% | 83.33% | 83.38% |
| **Empirical optimal cutpoint:** | 21.65 | 0.185 | 10.88 | 5.505 |
| **Sensitivity at cutpoint** | 0.69 | 0.66 | 0.72 | 0.7 |
| **Specificity at cutpoint** | 0.82 | 0.65 | 0.69 | 0.79 |
| **Area under ROC curve at cutpoint** | 0.75 | 0.65 | 0.7 | 0.74 |

**Serious bacterial infections**

|  | **CRP (mg/L)** | **PCT (ng/ml)** | **WBC (x10^9/L)** | **Neutrophil count (x10^9/L)** |
| --- | --- | --- | --- | --- |
| **OR** | 1.017(95% CI: 1,008 – 1.026, p < 0.001) | 1.130(95% CI: 1.062 – 1.203, p < 0.001) | 0.995(95% CI: 0.913 – 1.084 p < 0.001) | 0.999(95% CI: 0.991 – 1.008, p < 0.001) |
| **Sensitivity** | 0% | 22.22% | 0% | 0% |
| **Specificity** | 100% | 99.58% | 100% | 100% |
| **Positive predictive value** |  | 80% |  |  |
| **Negative predictive value** | 94.95% | 94.44% | 94.76% | 94.68% |
| **Empirical optimal cutpoint:** | 12.6 | 0.78 | 10.235 | 4.475 |
| **Sensitivity at cutpoint** | 0.67 | 0.67 | 0.38 | 0.57 |
| **Specificity at cutpoint** | 0.61 | 0.83 | 0.54 | 0.61 |
| **Area under ROC curve at cutpoint** | 0.64 | 0.75 | 0.46 | 0.59 |

**Urinary tract infections and Serious Bacterial Infections**

|  | **CRP (mg/L)** | **PCT (ng/ml)** |
| --- | --- | --- |
| **OR** | 1.035(95% CI: 1,025 – 1.045p < 0.001) | 1.162(95% CI: 1.054 – 1.280, p < 0.001) |
| **Sensitivity** | 29.81% |  |
| **Specificity** | 96.79% |  |
| **Positive predictive value** | 75.61% |  |
| **Negative predictive value** | 80.53% |  |
| **Empirical optimal cutpoint:** | 21.65 |  |
| **Sensitivity at cutpoint** | 0.64 |  |
| **Specificity at cutpoint** | 0.84 |  |
| **Area under ROC curve at cutpoint** | 0.74 |  |

**Multivariate models including CRP or PCT and clinical and laboratory data for viral infections diagnosis**

|  | **OR** | **Std. err.** | **z** | **P>z** | **[95% conf.** | **interval]** |
| --- | --- | --- | --- | --- | --- | --- |
|  |  |  |  |  |  |  |
| Age (days) | 1.021 | 0.007 | 3.101 | 0.002 | 1.008 | 1.035 |
| Fail thrive | 2.189 | 0.7 | 2.449 | 0.014 | 1.169 | 4.097 |
| rhinitis | 3.237 | 0.975 | 3.901 | <0.001 | 1.794 | 5.840 |
| dyspnea | 4.584 | 3.067 | 2.276 | 0.023 | 1.235 | 17.012 |
| Crp value | 0.98 | 0.009 | -2.100 | 0.036 | 0.962 | 0.999 |
| Positive urinalysis | 0.098 | 0.053 | -4.293 | <0.001 | 0.034 | 0.283 |

| **viral_inf** | **OR** | **Std. err.** | **z** | **P>z** | **[95% conf.** | **interval]** |
| --- | --- | --- | --- | --- | --- | --- |
|  |  |  |  |  |  |  |
| Age (days) | 1.024 | 0.009 | 2.845 | 0.004 | 1.008 | 1.041 |
| Fail thrive | 1.594 | 0.691 | 1.076 | 0.282 | 0.682 | 3.727 |
| rhinitis | 2.382 | 0.923 | 2.241 | 0.025 | 1.115 | 5.090 |
| dyspnea | 7.205 | 6.473 | 2.198 | 0.028 | 1.239 | 41.916 |
| Pct value | 0.665 | 0.248 | -1.093 | 0.274 | 0.32 | 1.382 |
| Positive urinalysis | 0.069 | 0.042 | -4.424 | <0.001 | 0.021 | 0.225 |

**Multivariate models including CRP or PCT and clinical and laboratory data for serious bacterial infections diagnosis**

|  | **OR** | **Std. err.** | **z** | **P>z** | **[95% conf.** | **interval]** |
| --- | --- | --- | --- | --- | --- | --- |
|  |  |  |  |  |  |  |
| Age (days) | 0.95 | 0.014 | -3.393 | 0.001 | 0.923 | 0.979 |
| Crp value | 1.014 | 0.005 | 2.695 | 0.007 | 1.004 | 1.024 |
| Positive urinalysis | 3.672 | 2.209 | 2.162 | 0.031 | 1.129 | 11.940 |

|  | **OR** | **Std. err.** | **z** | **P>z** | **[95% conf.** | **interval]** |
| --- | --- | --- | --- | --- | --- | --- |
|  |  |  |  |  |  |  |
| Age (days) | 0.927 | 0.021 | -3.387 | 0.001 | 0.888 | 0.969 |
| Pct value | 1.113 | 0.033 | 3.593 | <0.001 | 1.050 | 1.181 |
| Positive urinalysis | 3.791 | 2.895 | 1.744 | 0.081 | 0.848 | 16.940 |

**Multivariate models including CRP or PCT and laboratory data for urinary tract infections diagnosis**

|  | **Odds ratio** | **Std. err.** | **z** | **P>z** | **[95% conf.** | **interval]** |
| --- | --- | --- | --- | --- | --- | --- |
|  |  |  |  |  |  |  |
| Crp value | 0.998 | 0.005 | -0.493 | 0.622 | 0.988 | 1.007 |
| wbc | 1.103 | 0.034 | 3.208 | 0.001 | 1.039 | 1.170 |
| Positive urinalysis | 26.750 | 11.066 | 7.944 | <0.001 | 11.890 | 60.181 |

|  | **Odds ratio** | **Std. err.** | **z** | **P>z** | **[95% conf.** | **interval]** |
| --- | --- | --- | --- | --- | --- | --- |
|  |  |  |  |  |  |  |
| Pct value | 0.918 | 0.039 | -1.988 | 0.047 | 0.844 | 0.999 |
| wbc | 1.086 | 0.043 | 2.104 | 0.035 | 1.006 | 1.173 |
| Positive urinalysis | 35.507 | 17.153 | 7.389 | <0.001 | 13.776 | 91.521 |

**Multivariate models including CRP or PCT and clinical and laboratory data for urinary tract infections and serious bacterial infections diagnosis**

|  | **Odds ratio** | **Std. err.** | **z** | **P>z** | **[95% conf.** | **interval]** |
| --- | --- | --- | --- | --- | --- | --- |
|  |  |  |  |  |  |  |
| Age (days) | 0.982 | 0.009 | -1.927 | 0.054 | 0.964 | 1.000 |
|  |  |  |  |  |  |  |
| Maternal vaginal swab |  |  |  |  |  |  |
| Positive | 4.745 | 3.062 | 2.413 | 0.016 | 1.340 | 16.810 |
| Unknown | 0.786 | 0.342 | -0.553 | 0.58 | 0.336 | 1.842 |
|  |  |  |  |  |  |  |
| Crp value | 1.009 | 0.006 | 1.470 | 0.141 | 0.997 | 1.021 |
| Neutrophil count | 1.148 | 0.057 | 2.790 | 0.005 | 1.042 | 1.265 |
| Positive urinalysis | 28.777 | 12.851 | 7.523 | <0.001 | 11.993 | 69.049 |

|  | **Odds ratio** | **Std. err.** | **z** | **P>z** | **[95% conf.** | **interval]** |
| --- | --- | --- | --- | --- | --- | --- |
|  |  |  |  |  |  |  |
| Age (days) | 0.992 | 0.011 | -0.75 | 0.453 | 0.971 | 1.013 |
|  |  |  |  |  |  |  |
| Maternal vaginal swab |  |  |  |  |  |  |
| Positive | 3.303 | 2.322 | 1.700 | 0.089 | 0.833 | 13.097 |
| Unknown | 0.405 | 0.222 | -1.645 | 0.1 | 0.138 | 1.188 |
|  |  |  |  |  |  |  |
| pct_value | 1.037 | 0.033 | 1.132 | 0.258 | 0.974 | 1.104 |
| Neutrophil count | 1.095 | 0.062 | 1.604 | 0.109 | 0.98 | 1.224 |
| Positive urinalysis | 38.602 | 20.122 | 7.009 | <0.001 | 13.897 | 107.228 |


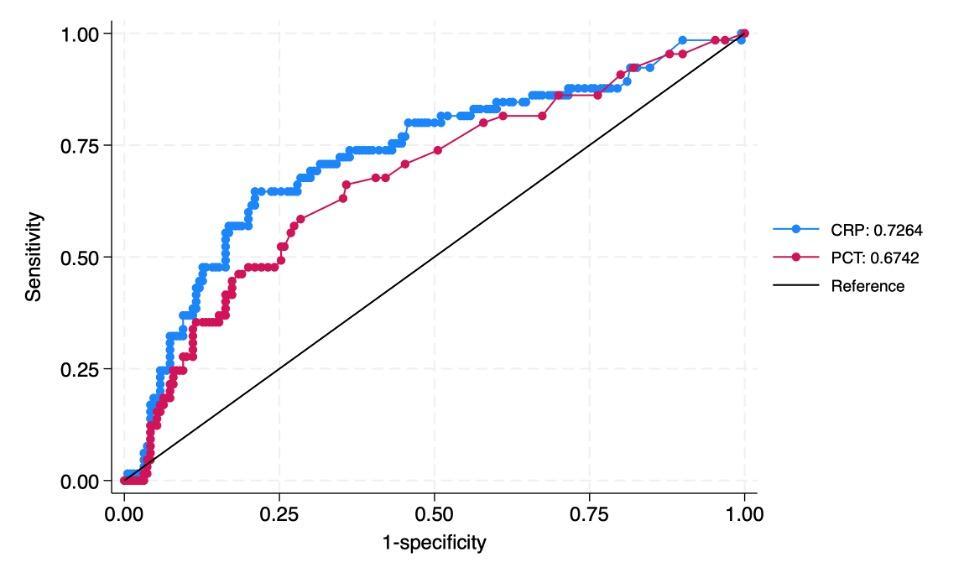


**A**


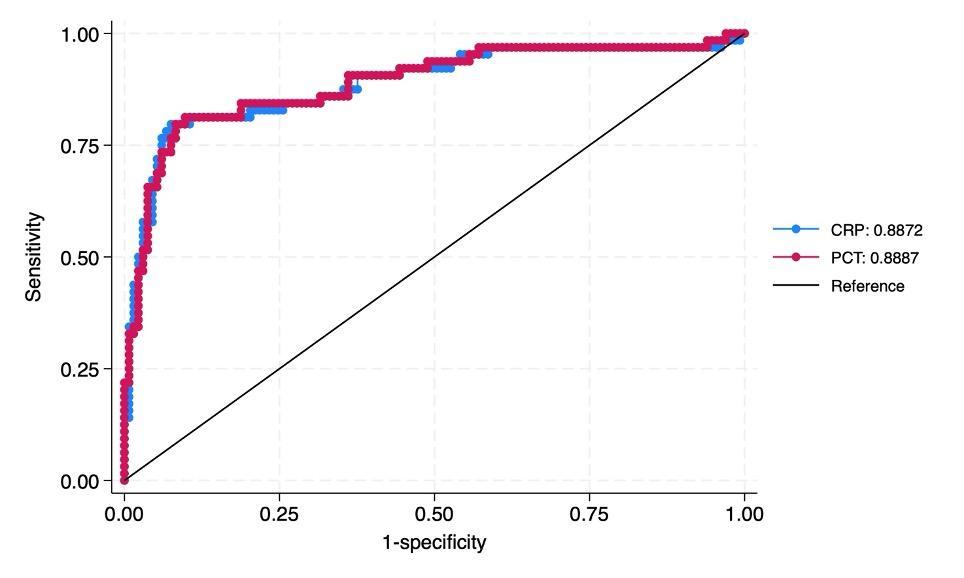


**B**

***Figure 1. Comparison of ROC curves for CRP and PCT alone (A) or combined with urine test (B) in discriminating UTIs.***


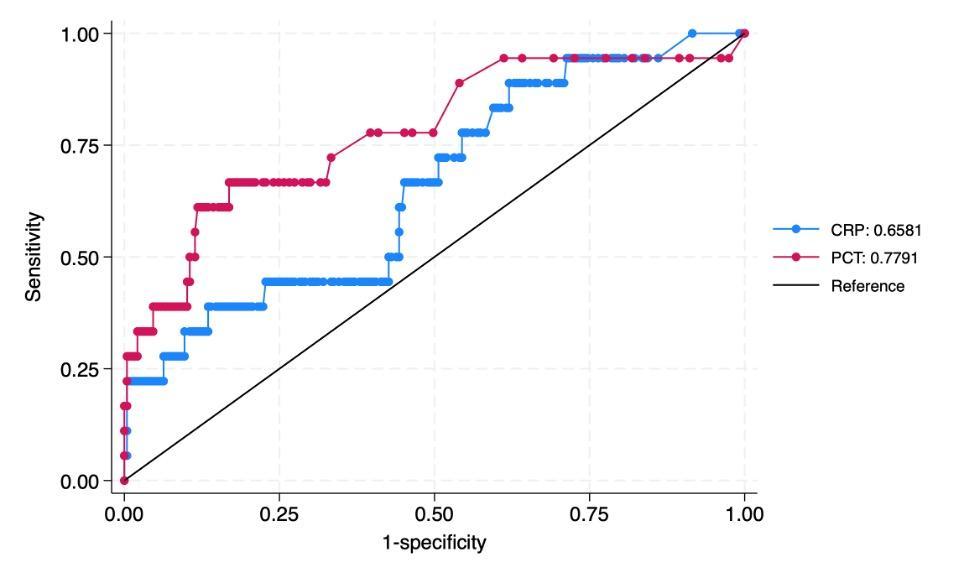


**A**


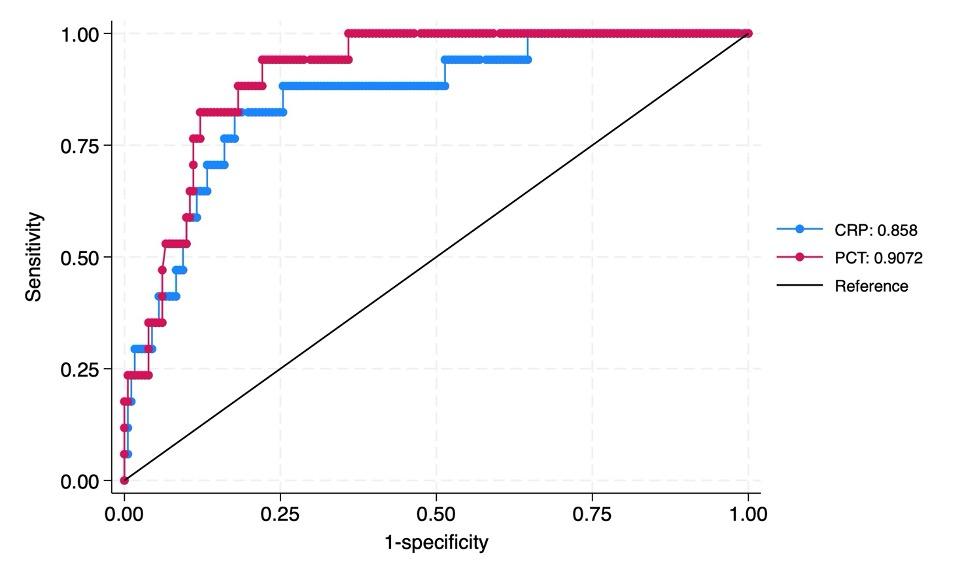


**B**

***Figure 2 Comparison of ROC curves for CRP and PCT alone (A) or combined with urine test (B) in discriminating SBIs***


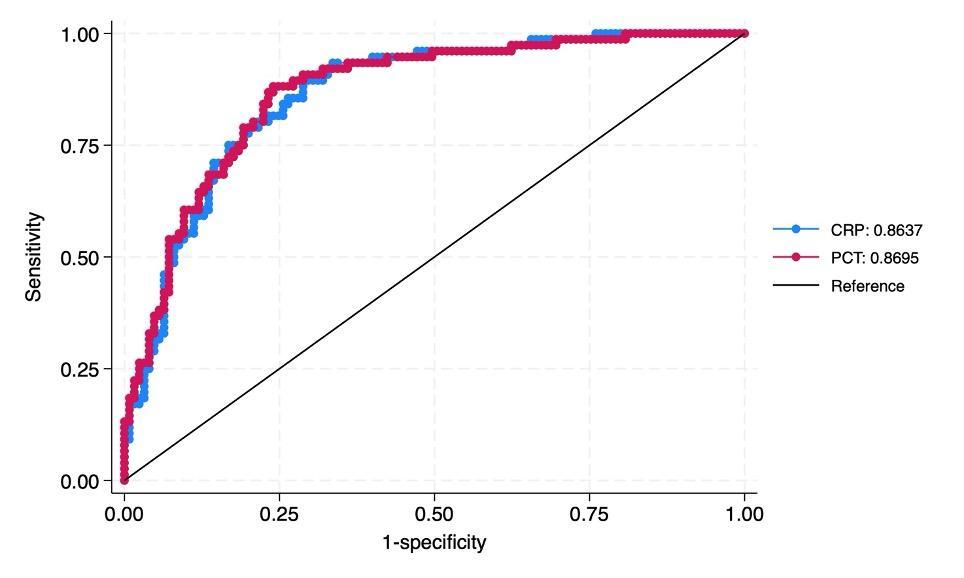


***Figure 3 Comparison of ROC curves for CRP and PCT with clinical factors and urine chemical test in discriminating viral infections.***
